# Lactate Gap: A Diagnostic Support in Severe Metabolic Acidosis of Unknown Origin

**DOI:** 10.1155/2018/5238240

**Published:** 2018-07-24

**Authors:** Linn E. Hauvik, Mercy Varghese, Erik W. Nielsen

**Affiliations:** ^1^Department of Anesthesiology and Critical Care Medicine, Nordland Hospital Bodø, Bodø, Norway; ^2^Department of Gastrointestinal Surgery, Nordland Hospital Bodø, Bodø, Norway; ^3^Institute of Clinical Medicine, University of Tromsø, Tromsø, Norway; ^4^Nord University, Bodø, Norway; ^5^University of Oslo, Oslo, Norway

## Abstract

Ethylene glycol poisoning is a medical emergency. The metabolites glycolate and glyoxylate give metabolic acidosis. Because of similar structure, these metabolites are misinterpreted as lactate by many point-of-care blood gas analyzers. The falsely high lactate values can lead to misdiagnosis, inappropriate laparotomies, and delayed antidotal therapy. As laboratory analyzers measure plasma lactate only, the difference or the “lactate gap” aids in early diagnosis. We present a patient with severe metabolic acidosis and elevated lactate levels on the point-of-care analyzer. A lactate gap supported the diagnosis of ethylene glycol poisoning. Hemodialysis and fomepizole treatment could be started immediately.

## 1. Introduction

Ethylene glycol poisoning is a medical emergency characterized by central nervous system depression, severe metabolic acidosis, cardiopulmonary complications, acute renal failure, and even death [1]. Metabolic acidosis is mainly caused by the ethylene glycol metabolite glyoxylic acid. The conversion of glycolic acid to oxalic acid leads to proximal tubular necrosis, a precursor to renal parenchymal damage and renal failure [2–4]. Treatment of ethylene glycol poisoning is time dependent, and early diagnosis is crucial to prevent morbidity and mortality [5]. This case report points to lactate gap as an early aid.

## 2. Case Description

A 60-year-old man with a history of alcohol abuse was found at home with impaired consciousness of unknown origin. The patient's room had numerous unlabeled cans of unknown contents. He was admitted to the hospital's intensive care unit (ICU).

**Figure 1:**
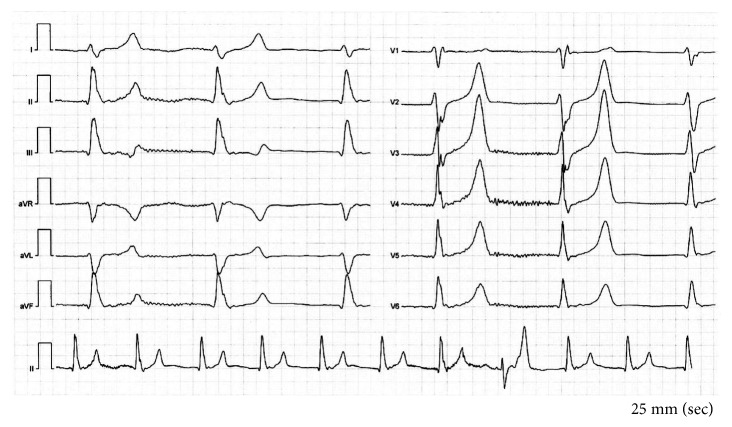
Electrocardiography (ECG) at time of admission. Elevated T-waves.

On arrival at the ICU, the patient had a Glasgow Coma Scale score of 3 (3–15). His pupils were equal, round, but nonreactive to light. His skin was pale and cool to touch, and rectal temperature was 32.7°C. The respiration was deep and quiet of 40–50 breaths per minute. The rest of the physical examination was unremarkable. Blood pressure was 144/60 mmHg, heart rate was 77 beats per minute, and the saturation on 3 L of oxygen was 100%. The electrocardiogram showed a supraventricular rhythm with elevated T-waves (Figure 1).

A point-of-care (POC) blood gas analysis in the ICU using Radiometer ABL800 FLEX blood gas analyzer (Medical Brønshøy, Denmark) showed pH of 6.77, pCO_2_ 1.5 kPa, pO_2_ 23.5 kPa, bicarbonate 2.0 mmol/L, and base excess −30 mmol/L. Serum potassium was 7.4 mmol/L, and lactate was so high that it was not measurable (Table 1). To prevent arrhythmia, intravenous calcium chloride was given prophylactically. Infusion of insulin-glucose and bicarbonate was started due to hyperkalemia and severe acidosis. Since no immediate clinical cause could be identified for the unexpected lactic acidosis, extended venous blood analyses and toxicological screening on blood and urine were performed. This revealed an osmolar gap of 106 mOsm/kg H_2_O, and anion gap was calculated to 41 mmol/L (Table 1). The urine sediment showed plenty of calcium oxalate crystals. Ethanol, paracetamol, and salicylate levels were negative. The plasma lactate level measured on the laboratory analyzer Vitros 5.1 (Ortho Clinical Diagnostics, Inc., Raritan NJ) was only 3.8 mmol/L. Repeated analyses confirmed high lactate levels measured on the POC blood gas analyzer and comparatively low lactate levels measured on the laboratory analyzer.

Taken together, the clinical and biochemical presentation was consistent with reports in the literature of a “lactate gap” [1, 3, 5–7]. From a diagnostic conundrum some years earlier [8], we had learned that the finding of a lactate gap indicates intoxication from ethylene glycol rather than methanol. A tentative diagnosis of ethylene glycol intoxication was made. Intravenous fomepizole therapy was initiated with a loading dose of 15 mg/kg. Hemodialysis was started two hours after admission due to persistent severe metabolic acidosis and electrolyte imbalance. Given the patient's extremely efficient respiratory compensation, it was decided not to intubate the trachea immediately.

After the patient's metabolic status improved, the trachea was intubated, and the patient mechanically ventilated as the depressed neurological state persisted. Intravenous fomepizole treatment continued during a 6-hour hemodialysis. Osmolar gap decreased to 7 mOsm/kg H_2_O after 12 hours. The patient regained consciousness the next day. He was transferred to a general ward after extubation of the trachea.

The patient developed anuric acute renal failure with a peak serum creatinine concentration of 872 *μ*mol/L for which he needed additional sessions of hemodialysis for a total of 11 days. On the 16th day, the patient was discharged from the hospital in his habitual condition. Not until the 35th day, creatinine (96 *μ*mol/L) and glomerular filtration rate (>60 mL/min/1.73 m^2^ based on creatinine, age, and sex) were within the normal reference area.

Our hospital does not have access to analysis of serum ethylene glycol nor methanol. A week after, results of toxicological screening of the blood at admission showed serum ethylene glycol level of 103 mmol/L (normally not in serum).

## 3. Discussion

**Figure 2:**
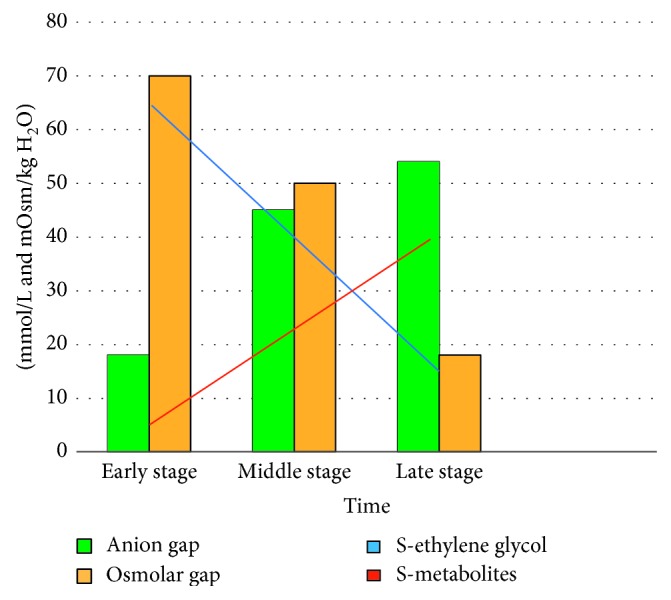
Anion and osmolar gap as metabolism of ethylene glycol progresses (redrawn after Hovda et al. [11]).

The diagnosis of ethylene glycol poisoning is challenging due to nonspecific signs and symptoms [3]. The initial presentation of ethylene glycol poisoning depends on the time elapsed since ingestion, amount ingested, and coingestion of alcohol dehydrogenase inhibitors (Figure 2) [2].

The definitive laboratory test for ethylene glycol poisoning is ethylene glycol serum concentration. This test is not readily available in all laboratories and may significantly delay diagnosis and treatment in a situation where time is of the essence [2, 3, 5]. Additionally, patients presenting late after intake may have normal ethylene glycol levels (Figure 2). They could be critically ill, however, having metabolized ethylene glycol to its toxic metabolites already (Figure 2) [9].

The lactate gap between an arterial blood gas and a venous blood sample is therefore mainly the presence of ethylene glycol metabolites glycolate and glyoxylate, which are cross-reacting with the point-of-care analyzer.

**Figure 3:**
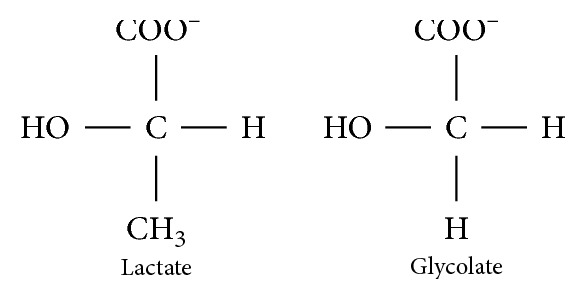
The chemical configuration of lactate and glycolate (redrawn after Morgan et al. [7]).

When faced with a patient who exhibits a combination of osmolar gap and anion gap acidosis, ethylene glycol or methanol poisoning should be considered early on. A third “gap,” the lactate gap is a phenomenon of great clinical significance and utility. In several studies, it has been noted that lactic acid measurements may be severely but falsely elevated in ethylene glycol intoxication, depending on the type of analyzer used. In particular, most POC whole blood analyzers which use L-lactate oxidase cross-react with glycolate or glyoxylate, breakdown products from ethylene glycol (Figure 3), leading to large false lactate elevations [5, 7]. In contrast, several laboratory serum analyzers, which are used for routine analysis of venous blood samples, have less cross-reactivity and show minimal lactate elevation [10]. The combination of POC and laboratory analyzers is therefore important to show a lactate gap. Conversely, absence of a lactate gap does not rule out ethylene glycol poisoning. Measuring the lactate gap can be exploited for diagnosis of ethylene glycol poisoning, even in late presentations, as well as for monitoring clearance of glycol metabolites [9].

If not known, this falsely elevated lactate level can lead to a misdiagnosis of lactate acidosis, can delay antidotal therapy and even justify inappropriate surgeries such as laparotomies [5]. Knowledge of the analytical interference of glycolate and glyoxylate leading to false lactate elevation on many POC blood gas analyzers is therefore crucial when seeing patients with severe metabolic acidosis and massive lactate elevation [6].

## 4. Conclusion

We present a patient with severe anion gap metabolic acidosis with pH 6.77, with an elevated osmolar gap and high lactate levels. Treatment with fomepizole and hemodialysis lead to a successful outcome. Our finding of a “lactate gap” hastened the diagnosis of ethylene glycol poisoning, avoided misdiagnosis, and prevented delay in treatment.

## Figures and Tables

**Table 1:** Laboratory values.

Measurement	Arrival at ICU	8 hours later	Reference values
Lactate, blood (POCT)^*∗*^	Higher than upper measuring limit	10.7	<2 mmol/L
Lactate, plasma^*∗∗*^	3.8	1.3	<2 mmol/L
pH^*∗*^	6.77	7.37	7.37–7.45
pCO_2_^*∗*^	1.5	6.9	4.7–6.0 kPa
Bicarbonate^*∗*^	2	29	22–27 mmol/L
Base excess^*∗*^	−30	4	0 ± 3
Potassium^*∗*^	7.4	4.3	3.5–5.0 mmol/L
Anion gap^1^	41	17	3–9 mmol/L
Osmolality^*∗∗*^	426	306	280–300 mOsmol/kg H_2_O
Osmolar gap^2^	106	2	<10 mOsmol/kg H_2_O

^*∗*^Measured with a Radiometer ABL800 FLEX point-of-care whole blood analyzer (bicarbonate and base excess are calculated). ^*∗∗*^ Measured with a Vitros 5.1-plasma laboratory analyzer. ^1^Anion gap = ([Na^+^] + [K^+^]) − ([Cl^−^] + [HCO_3_^−^]). ^2^Osmolar gap = measured osmolality − (1.86 × [Na^+^] + [glucose] + [urea]/0.93).
